# Testing different function fitting methods for mobile eye-tracker calibration

**DOI:** 10.16910/jemr.16.4.2

**Published:** 2023-09-14

**Authors:** Björn R. Severitt, Thomas C. Kübler, Enkelejda Kasneci

**Affiliations:** Eberhard Karl University of Tübingen, Germany; Look! ET, Germany; Human-Centered Technologies for Learning, Technical University of Munich, Germany

**Keywords:** Gaze Estimation, Gaze vectors, Simulation, Regression, Calibration, Eye Tracking

## Abstract

During calibration, an eye-tracker fits a mapping function from features to a target gaze point. While there is research on which mapping function to use, little is known about how to best estimate the function's parameters.

We investigate how different fitting methods impact accuracy under different noise factors, such as mobile eye-tracker imprecision or detection errors in feature extraction during calibration. For this purpose, a simulation of binocular gaze was developed for a) different calibration patterns and b) different noise characteristics.

We found the commonly used polynomial regression via least-squares-error fit often lacks to find good mapping functions when compared to ridge regression. Especially as data becomes noisier, outlier-tolerant fitting methods are of importance. We demonstrate a reduction in mean MSE of 20% by simply using ridge over polynomial fit in a mobile eye-tracking experiment.

## Introduction

The calibration of an eye tracker is the challenge of mapping features extracted from an image of the eye onto the scene camera's view to obtain the point of regard. An established method is to derive two polynomials, one for the x-coordinate and one for the y-coordinate pixel. Commonly used features are the pupil centre, the vector between pupil and corneal reflection or the eyeball orientation. The workflow is to collect correspondences between eye features and a known target point that the person looks at. From these associated features and gaze target coordinates an estimation of the coefficients of a polynomial can be performed. The estimation is done via least-squares-error fitting.

In a desktop eye tracking scenario, n-point calibration is the state of the art because it is easy to visualise points on the screen and get the subject to look at them. In a head-mounted scenario, there is no screen available, so the subject must look at a marker that can be easily detected. The marker is then moved over the scene field.

While both methods seem to produce similar data correspondences, there are some major differences in practice: In a desktop scenario, the recorded points are evenly distributed over the screen. These points span the entire screen and thereby the entire target area. False measurements can be compensated by averaging over all samples associated with a specific gaze target.

**Figure 1.**
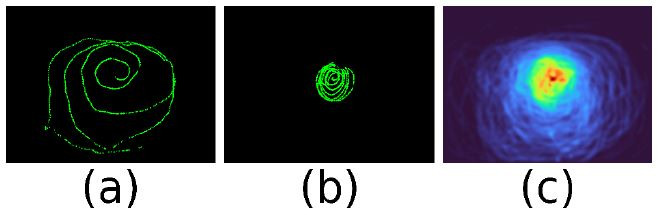
Smooth pursuit calibration of a mobile eye-tracking device. A gaze target marker is followed by the eye and moved in front of the scene camera. The first image is from Subject 1 and covers a large area. The second is the result of Subject 2 and covers only the central area. The third is a density map over 7 subjects.

In the head-mounted scenario the distribution of samples over the scene field depends on how the subject performs the calibration. The images in Figure 1 (a) and (b) shows calibration paths of two subjects with identical instructions on how to perform the calibration. While the first calibration was done over a large field of the scene but also with a high density towards the centre, the second one calibrated the centre only. We found that most often sample density is high towards the centre and low towards the periphery. This is problematic when applying least-squares-error fitting to the problem, as the central area will likely dominate over the less important and consequently less accurate periphery data.

Moreover, large gaze angles are often completely avoided or performed with simultaneous head rotation. The resulting implication for a calibration function is a need to extrapolate gaze outside of the calibrated area.

Overall, the gaze signal in mobile eye-tracking suffers from much less constrained conditions as in the desktop case, often resulting in decreased quality of the gaze signal and more false measurements - especially towards large gaze angles, where the pupil or glints are harder to track. In general, mismeasurements may be due to incorrect pupil or glint detection, environmental influences such as brightness or reflections in the pupil. There are a variety of sources of mismeasurement. As we cannot always easily exclude these samples from the calibration, the way we fit the calibration function must be tolerant to outliers.

The image of Figure 1 (c) shows a heat map over seven recorded calibration paths. Again, the problem of different sample density is obvious. Fitting a calibration function has to cope with unbalanced sample densities, areas that are not covered, and outliers in the eye features data.

A least-squares error fit of a high degree polynomial is likely to yield unpredictable results for extrapolation of gaze outside of the calibrated area, while a low degree polynomial might lead to less accurate results within the calibrated area.

In order to investigate which fitting methodology is well suited for which purpose, we need to investigate the effect of different measurement error characteristics as well as different calibration patterns on the resulting calibration. To be able to vary their parameters in a controlled and quantified way, we performed:

- A simulation of gaze vectors during simulated calibration. Horizontal and vertical angles between the optical axis and the line from the centre of the eyeball to the eye camera are simulated and enriched with controlled patterns of measurement noise. We use these gaze angles because they have proven to be relatively stable in terms of device slippage (18).
- A simulation of different calibration patterns. Different target patterns (e.g., circular pursuit, 9-point calibration) are investigated in different conditions. In particular, the difference in performance for inter- and extrapolation is addressed.
- To transfer the results of the simulation to the real world, we created a real-world study where we recorded calibration data of 7 subjects. So, we obtain real calibration patterns, and it gives us the opportunity to confirm that the simulated results can be transferred to a real application. In this case, we compare the performance of the calibration method with the simulated data of the real samples with the data from the experiment.

## Related Work

As essential building blocks of an eye-tracking device, calibration functions have been studied in some detail - even though other components such as pupil detection have experienced much more attention by the community (8).

For example, Kasprowski et al. (11) analysed possible scenarios of different simulation presentations and discussed the influence of different regression functions and two different head-mounted eye trackers on the results. Ultimately, however, they cannot say which regression is the best, because the performance is different across different eye trackers. Accordingly, they advise that the regression function used should be optimised for the eye tracker, raising thus the question on which properties of the device make the difference.

Using the pupil centre - corneal reflection vector as an input feature was found to make the calibration somewhat resilient to head movements. This approach is explored in more detail by Blignaut (2). The author optimised the calibration configuration of the hardware configuration and tested several mapping functions.

In addition, Blignaut proposed a method for identifying a set of polynomial expressions that provide the best possible accuracy for a specific individual (3). Real-time recalculation of regression coefficients and real-time gaze correction are also proposed. In the evaluation, Blignut concludes that the choice of polynomial is very important for accuracy when no correction is used. However, when real-time correction is used, the performance of each polynomial improves, while the choice of polynomials becomes less critical.

The influence of the placement of the eye camera on the results was also investigated. Narcizo et al. showed that the distribution of the features of the eyes is deformed when the eye camera is far away from the optical axis of the eye (15). To solve this problem, they propose a geometric transformation method to reshape the distribution of eye features based on the virtual alignment of the eye camera at the centre of the optical axis of the eye. That leads to a high gaze estimation accuracy of 0.5.

Recently. Hassoumi et al. improve the calibration accuracy by a symbolic regression approach (9). Instead of making prior assumptions about the polynomial transfer function between input and output data sets, this approach seeks an optimal model from different types of functions and their combinations. The authors achieved a substantial 30% improvement in calibration accuracy compared to previous approaches.

A similar work to ours is that of Drewes et al. (5). They tried two circular trajectories with different radii. Both were run on a display in front of which the participants sat. In each calibration, an offset, a regression, and a homography calibration were tried, and it was found that the accuracy of offset and regression was similar for the circular trajectory, and the precision of regression was better. The homography approach is the worst in both cases.

Although there are several related works that have addressed calibration accuracy, but rather few have on gaze vectors. Therefore, in this paper we have focused on mapping these features onto the scene using well-known methods such as polynomial regression.

Summarised, previous research underpins the importance of research in how and which calibration functions should be used. There is no clear consensus on the optimal function and how that function is fitted is probably of similar importance as the function itself. Likely, the magnitude of measurement errors as well as the nature of samples collected during the calibration process drive this decision.

## Dataset

Two data sets are used in this work. One of these data sets is simulated, the other contains real-world recordings of smooth pursuit calibration processes of a head-mounted device.

### Simulated Data

This dataset consists of simulated gaze vectors and the associated targets. Gaze vectors are the horizontal and vertical angle between the optical line and the line from the centre of the eyeball to the eye camera. In a more formal way: Let $c_{eye}\in {\mathbb{R}}^{3}$ be the centre of the eye and $e_{cam}\in {\mathbb{R}}^{3}$ the position of the eye camera, than

$$ce=e_{cam}-c_{eye}\in {\mathbb{R}}^{3}$$

Is the direction vector from the centre of the eye to the eye camera. Furthermore, let $o\in {\mathbb{R}}^{3}$ be the direction vector of the optical axis (see Figure 3). In our scenario, the z-axis is the depth, the y-axis is the height, and the x-axis is the width. The horizontal angle is therefore the rotation around the y-axis, i.e., the angle in the x-z plane, and the vertical angle is the rotation around the x-axis, i.e. the angle in the y-z plane. So, the horizontal angle can be calculated as follows: Let $ce_{hori},\;o_{hori}\in {\mathbb{R}}^{2}$ be the direction vectors without the y-component. Then the horizontal angle ϕ can be calculated as follows:

$$\phi =arccos\left(\frac{ce_{hori}^{T}\cdot o_{hori}}{∥ce_{hori}∥\cdot ∥o_{hori}∥}\right)$$

The vertical angle θ can be calculated in a similar way, with $ce_{vert},o_{vert}\in {\mathbb{R}}^{2}$ being the direction vectors without the x-components. The gaze vector is thus defined as:

$$gv≔\left(\begin{array}{c} \phi \\ \theta \end{array}\right)$$

**Figure 2.**
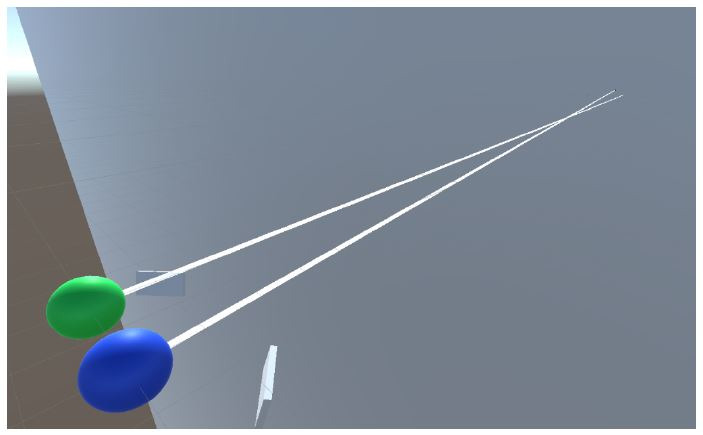
Unity 3D scene that represents objects involved in the simulation.

To simulate gaze vectors, we used Unity with the scripting language C#, to cover all the heavy lifting with 3D geometry as well as visualization. A brief explanation of the use of Unity in this work can be found in the appendix. In contrast to eye image synthesis (14), (12), (20), our purpose does not necessitate a full eye model. The representation as a simple sphere is fully sufficient and numerical output is much faster to calculate than graphical renderings. We created a scene with two spheres that represent the eyes, a plane with a point that serves as a fixation point, and dummies for the eye cameras. Figure 2 shows the scene.

Additionally, there is a camera over the two spheres, producing the cyclops image of an eye-tracker's field camera.

To create the gaze vectors, we move a fixation target on a plane in front of the eyes. The position is determined via the field camera. To achieve this, we specify two values x,y∈[0,1], which indicate the distance between the left bottom edge of the image and the target point. Then we rotate the two spheres so that the optical axes (the line through pupil centre and cornea centre) are directed towards the point.

**Figure 3.**
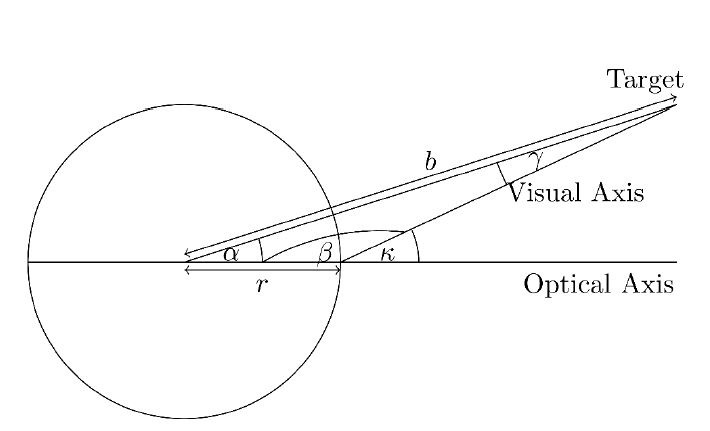
Sketch for calculating the angle of rotation α to simulate a given κ.

To include an individual offset between optical and visual axis (the line through pupil centre and fovea), we need to simulate the κ angle. We simulate a horizontal and vertical angle: $\kappa_{hori},\;\kappa_{vert}$. Since the spheres can only be rotated at their centre, we have to calculate this angle. Figure 3 shows a sketch of the situation.

Given κ we search for the angle α. We know the radius r of the sphere and the distance b between the centre of the sphere and the target. In addition, the β angle can be represented as follows:

β=180−κ

Thus follows:

α+β+γ=180⇒α+(180−κ)+γ=180

⇒γ=κ−α

With the law of sines, we get:

$$\frac{r}{\sin\gamma }=\frac{b}{\sin\beta }\Leftrightarrow \frac{r}{\sin\left(\kappa -\alpha \right)}=\frac{b}{\sin\left(180-\kappa \right)}$$

When this is transformed to α, it follows:

$$\alpha =\kappa -\arcsin\left(\frac{r}{b}\cdot \sin\left(180-\kappa \right)\;\right)$$

With this formula and the given angles $\kappa_{hori}$ and $\kappa_{vert}$ we create $\alpha_{hori}$ and $\alpha_{vert}$ to rotate the sphere. We apply this transformation to both spheres.

To calculate the gaze vectors, a line is constructed from the centre of the dummy eye camera to the centre of the sphere. Then the horizontal ( ϕ) and vertical ( θ) angles between the constructed line and the optical line are calculated. These correspond to the gaze vectors.

**Figure 4.**
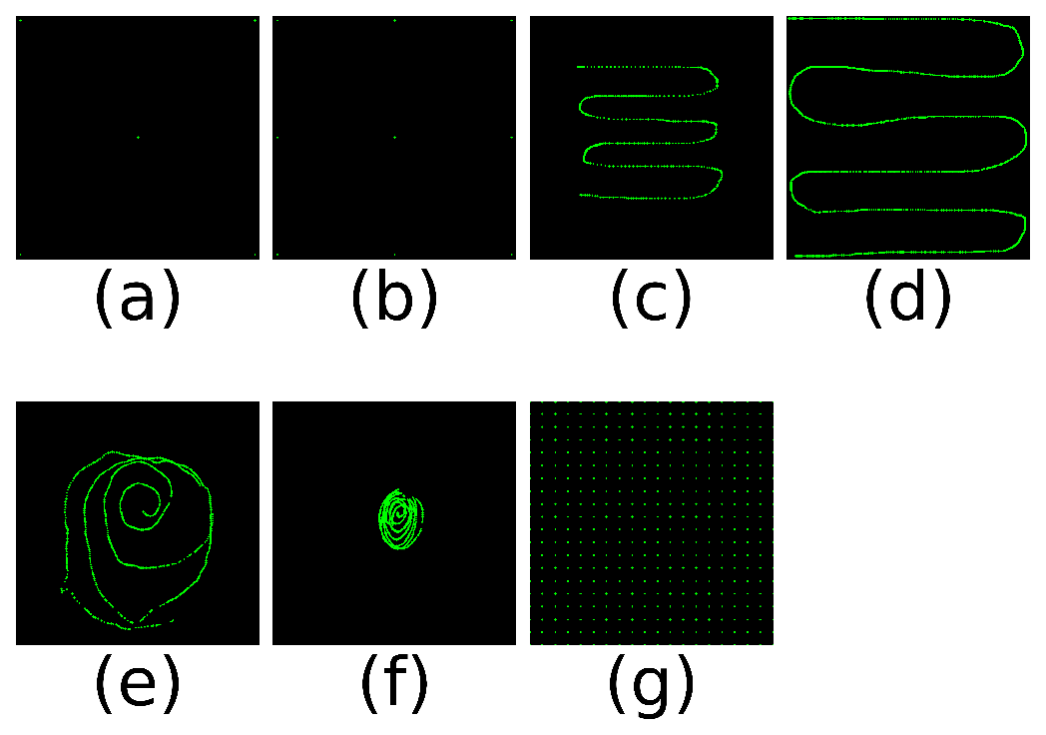
Pattern. (a) 5-point calibration (b) 9-point calibration (c) Centre calibration (d) Full calibration (e) Subject huge (f) Subject small (g) 20×20 full field

We simulate these gaze vectors for all positions within the calibration patterns shown in Figure 4.

We used the gaze vectors simulated on those patterns to estimate an eye-tracker calibration. Afterwards, we test the quality of the calibration on a test pattern that covers the whole field of view.

Moreover, we also simulated gaze vectors for calibration patterns found in the real-world experiment. This way, we can compare the simulation results directly to real-world observations. Below is a brief description of the patterns.

**5-point calibration (5p)** This is a normal 5-point calibration pattern with dots in the corners and one in the centre. It is usually used to fit a homography for gaze mapping.

**9-point calibration (9p)** This is a normal 9-point calibration pattern with three points on three lines. Popular for calibrating desktop eye-tracking devices.

**Centre calibration (Centre)** Snake pattern, simulating a smooth pursuit calibration, in the centre of the seen field.

**Full calibration (Full)** Snake pattern across the entire field of view.

**Subject huge (huge)** This pattern is extracted from the experiment to compare the simulation with the real data. This pattern covers almost the field.

**Subject small (small)** This pattern is extracted from the experiment to compare the simulation with the real data. This pattern covers only the centre field.

𝟐𝟎×𝟐𝟎 **full field** This is a pattern consisting of 20×20 dots evenly distributed over the entire field. It is used to evaluate the performance of calibrations.

Normally, the n-point calibration points tend to be centered because that is where the corners of the stimulus region of interest are. Since we are looking at the performance of the entire image, we placed the calibration points very close to the edges.

After simulating the error-free gaze vectors, we use Python to simulate three types of measurement noise: systematic error, precision noise, and completely misidentified samples.

The systematic error depends on the gaze vectors. The eye-tracking system we used to compare our results shows difficulties in determining reliable gaze vectors when the person looks into or close to the eye cameras. The detected pupil outline then becomes almost circular, making it difficult to tell where the person is looking at. For this reason, we added an error that is larger when the gaze vectors are small. For this purpose, we extracted the largest horizontal $\phi_{\max}$ and vertical $\theta_{\max}$ angle magnitude. With probability $p_{error}$, we apply an error to the gaze vector. When an error occurred the new gaze vector $\left(\phi_{sys},\;\theta_{sys}\right)$ is created as follows:

$$\phi_{sys}=\phi +{\left(\;\frac{2-\;\frac{\left|\phi \right|}{\phi_{\max}}-\;\frac{\left|\theta \right|}{\theta_{\max}}}{2}\right)}^{4}\left(u-0.5\right)r_{error}\pi$$

$$\theta_{sys}=\theta +{\left(\;\frac{2-\;\frac{\left|\phi \right|}{\phi_{\max}}-\;\frac{\left|\theta \right|}{\theta_{\max}}}{2}\right)}^{4}\left(u-0.5\right)r_{error}\pi$$

Where $r_{error}$ is a parameter for the maximum error and u∼U(0,1) is a uniformly distributed random number. If no error occurred, the gaze vectors remain unchanged.

The precision error is white noise $\epsilon_{prec}∼N\left(0,\;\sigma_{prec}\right)$ added to the gaze vectors (given in radians). The $\sigma_{prec}$ is a parameter with which we can simulate different precisions. This error can be considered a recording device specific. The new gaze vector $\left(\phi_{prec},\;\theta_{prec}\right)$ is created as follows.

$$\phi_{prec}=\phi_{sys}+\epsilon_{prec}$$

$$\theta_{prec}=\theta_{sys}+\epsilon_{prec}$$

In Table 1, we provide some values on how σ and the resulting magnitude of gaze angle deviation relate to each other. We show the 95% confidence intervals of added error in radians and degree.

**Table 1.** 95% confidence intervals for different *𝜎_prec_*

*𝜎_prec_*	**Radians**	**Degree**
0.001	±0.002	±0.112
0.005	±0.01	±0.561
0.01	±0.02	±1.123
0.05	±0.098	±5.615

The third type of error simulates when the pupil is detected in the wrong place for a few frames. This can happen due to several reasons, e.g., when an object is reflected in the eye, or the pupil is partially covered. For each gaze vector and per eye, we decided with a probability $p_{fd}=0.005$ whether a false detection has happened. If so, we choose a random number n∈[1,9]⊂ℕ to determine how many gaze vectors are corrupted. The final gaze vector $\left(\phi_{f},\theta_{f}\right)$ is calculated as follow, when a false detection occurred.

$$\phi_{f}=\phi_{prec}+\left(u-0.5\right)\cdot \frac{\pi }{2}$$

$$\theta_{f}=\theta_{prec}+\left(u-0.5\right)\cdot \frac{\pi }{2\;}$$

When no false detection occurred, the gaze vectors remain unchanged.

**Figure 5.**
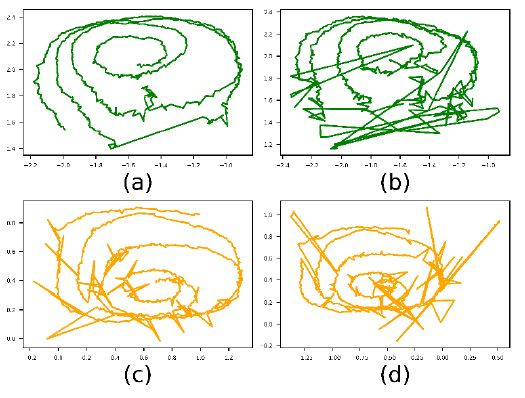
Example of real and simulated gaze vectors. The parameters for the simulated vectors are: $p_{error}=0.1,\;r_{error}=0.5$ and $\sigma_{prec}=0.005$. (a) and (b) are the left and right vectors from a real experiment and (c) and (d) are the left and right simulated ones.

An example of simulated gaze vectors with added measurement noise as well as a real recording is shown in Figure 5.

### Data collected in real-world experiment

For the experimental data collection, we used a head mounted eye-tracker by Look! (13), which operated at 30 Hz at an eye image resolution 320×240 px. The scene is recorded at 30 Hz and at a resolution of 640×480 px. To get the gaze vectors, we used the *Purest* pupil tracking method and the *Get a Grip* eye model (17), as implemented in EyeRecToo (17).

For the eye-tracking recording, subjects (2 females, 5 males, 16-36 years old, without glasses) were instructed to direct their gaze at a target marker. Subjects were instructed to keep their heads still and move only their eyes while following the 15×15 cm target aruco-marker. In the appendix you will find an example of an aruco-marker. They were instructed to move the marker in a large spiral from the centre outwards. This procedure is repeated once so that we have different data for estimating and evaluating the calibration.

Not all subjects followed the instructions as intended. Since this dataset is mainly intended to test the transferability of the simulated results to the real world, we allowed them to deviate slightly from the protocol to match the realistic expected calibration data.

This dataset is created to extract realistic calibration patterns (Subject small and huge see Figure 4e and f) and to provide proof of concept for the transferability of the results produced with the simulation to the real world. The number of subjects is too small to make statistically significant statements.

## Methods

Since Drewes et al. show in their paper (5) that regression works very well for circular calibration, we tested different ways to fit a calibration. All of them were implemented in the Python module scikit-learn (16) are briefly summarised in the following. The methods find a polynomial of degree n

$$p\left(x,\;\gamma \right)=\sum_{i=0}^{n}\gamma_{i}\cdot x^{i}$$

by minimising a loss function. The most trivial (and most often used) method is polynomial regression, which minimises the residual sum of squares

$$sse\left(\gamma \right)=\sum_{k=1}^{m}{\left(p\left(x_{k},\;\gamma \right)-y_{k}\right)}^{2}$$

Where $x_{k}$ are the features and $y_{k}$ the true value. In this method we have one parameter, namely the degree ( d=1,2,3,4,5) of the estimated polynomial. We have also tried **Lasso** (19) and **Ridge** (10) regression. They are very similar to polynomial regression, with the only difference being that the sum of the coefficients is added to the loss function, as shown below.

$$loss_{Lasso}\left(\gamma \right)=sse\left(\gamma \right)+\alpha \sum_{i=1}^{n}\left|\gamma_{i}\right|$$

$$loss_{Ridge}\left(\gamma \right)=sse\left(\gamma \right)+\alpha \sum_{i=1}^{n}\gamma_{i}^{2}$$

α>0is a weight parameter of the additional loss term. We performed a grid search over the degree of the polynomial and $\alpha =10^{-4},\;10^{-3},\;10^{-2},\;0.1,\;1,\;2$.

The above methods however do not explicitly cover outliers (such as false pupil detections). **RANSAC** (random sample consensus) (7) is an iterative algorithm that randomly splits the dataset into inliers and outliers and fits the model, in our case a polynomial regression, to the inlier dataset. Here we have only used the degree d=1,2,3. Since only a subset of the data points is used to estimate the polynomial, a larger data set is needed for the estimation. The patterns with 5 and 9 points (Figure 4 (a) and 4 (b)) do not have enough data points for a higher degree.

**Support Vector Regression** (SVR) (6) is an extension of the Support Vector Machine (SVM) (4). While the SVM looks for a hyperplane $f\left(x\right)=w^{T}\;\phi \left(x\right)+b$ that separates the classes in such a way that no point lies within a given margin, the SVR looks for a hyperplane where all points lie within the margin. In this case $\phi :\;{\mathbb{R}}^{d_{1}}\to {\mathbb{R}}^{d_{2}},\;d_{1}<d_{2}$ is a function that transforms a point into a higher dimensional space where the classes are linearly separable. To find this hyperplane, the optimization problem

$\min_{w,\;b,\;\xi ,\xi^{*}}$ $\frac{1}{2}w^{T}w+C\sum_{i=1}^{n}\left(\xi_{i}+\;\xi_{i}^{*}\right)$

subjectto $y_{i}-f\left(x_{i}\right)\leq \epsilon +\xi_{i},f\left(x_{i}\right)-\;y_{i}\leq \epsilon +\xi_{i}^{*},\xi_{i},\;\xi_{i}^{*}\geq 0,\;i=1,\ldots ,n$

is solved. Here ϵ is the margin, C is a weighting parameter for the penalty if a point is not within the margin and n is the count of training vectors. Since the optimisation problem is solved by the dual form, the hyperplane can be given as $f\left(x\right)=\;\sum_{i=1}^{n}a_{i}y_{i}\left\langle \phi \left(x_{i}\right),\;\phi \left(x\right)\right\rangle +b$, where $\left\langle \phi \left(x_{i}\right),\;\phi \left(x\right)\right\rangle$ is the scalar product of the higher dimensional transformation of $x_{i}$ and x and $a_{i}$ are the associated Lagrangian variables of the dual problem. Because of the high computational cost of this scalar product, a positive definite kernel function $K\left(x_{i},\;x\right)=\;\left\langle \phi \left(x_{i}\right),\;\phi \left(x\right)\right\rangle$ is used. In this work we tried three different kernel functions:

 linear: $K\left(x^{\prime },\;x\right)=\;\left\langle x^{\prime },\;x\right\rangle$

 polynomial: $K\left(x^{\prime },\;x\right)=\;{\left(c\left\langle x^{\prime },\;x\right\rangle \right)}^{d}$

 rbf: $K\left(x^{\prime },\;x\right)=exp\left(-c∥x^{\prime }-x∥\right)$

Here c=1/(4⋅Var(X)), where 4 is the number of different features and Var(X) is the variance of the feature matrix. This is the default of the *scikit-learn* module. For the degree d, we tried 1,2,3,4 and 5. In addition, we used for C the values $10^{-5},\;10^{-4},\;10^{-3},10^{-2},\;0.1,\;\;0.5,\;1,\;2,\;5$ and for ϵ the values $10^{-4},\;10^{-3},\;10^{-2},\;0.1,\;0.2,\;0.5,\;1$, respectively. Table 2 shows a summary of the methods used and their corresponding parameters, for which we also used different values.

**Table 2.** Methods used for fitting a calibration function as well as their parameters investigated.

**Method**	**Full Name**	**Parameter**
Poly	Polynomial Regression	(degree,)
Lasso	Lasso Regression	(degree, alpha)
Ridge	Ridge Regression	(degree, alpha)
RANSAC	Random Sample Cosensus Regression	(degree,)
SVR	Support Vector Regression	(kernel, degree, C, 𝜖)

In summary, all proposed methods have a different approach to estimate the function. Lasso and Ridge penalize the sum of the estimated coefficients, so smaller coefficients are preferred by these methods, which leads to the fact that fluctuations in the input values lead to less large fluctuations in the result, so the possibility to overfit should be smaller. Whereby Ridge penalizes coefficients smaller than one less and greater than one more than Lasso, because Ridge uses the sum of squares and Lasso the sum of magnitudes as penalty term. In both cases it is possible to control the importance of the penalty with the alpha-parameter.

RANSAC tries to identify outliers by dividing the input values several times and not to include them in the estimation of the polynomial. Therefore, this method should handle false measurements like missmatches well and identify that as outlier and not include them to the estimation.

SVR is the only method in this paper that estimates a hyperplane rather than a polynomial. This hyperplane is estimated in such a way that it tries to match all points as closely as possible. To prevent overfitting, the method has two hyperparameters. The first is ϵ, which specifies how far the estimated hyperplane is allowed to miss the points, and the second is C, which specifies the weighting of the penalty if a point is missed further than the allowed ϵ.

To evaluate the performance of the different methods, we created 100 different κ-angles with horizontal $\kappa_{hori}∼N\left(3.9,\;2.2\right)$ and vertical $\kappa_{vert}∼N\left(0.2,\;1.7\right)$ (1) for both eyes. With each κ-angle, we create a simulation for each pattern. Now the following steps are carried out for each calibration pattern:

1. Calculate the error for each simulation:
  - Fit the method with the gaze vectors to the target points of the calibration pattern used.
  - Estimate the gaze points with the gaze vectors of the full field pattern (Figure 4 (g))
  - Calculate the angle between the vector from the camera to the estimated point and the vector from the camera to the true point.
  - Calculate the mean of the amount of the angles. This is the mean angle error of the simulation.
2. Calculate the mean of the mean error angles of all simulations.

Note that the true and estimated points are within the camera image. They indicate the proportion of the vertical and horizontal axis of the image. To calculate the angle, the points must be transformed into the scene coordinates. The formula used can be found in the appendix.

**Table 3.** Best results for different precision qualities with the different calibration methods with the simulated gaze vectors. The standard deviation is given in the parentheses.

**Precision Error**	**Calibration**	**Method**	**Parameter**	**Mean Error (°)**
0.001	5-point	Lasso	(3, 0.001)	2.718 (±1.184)
	9-point	Ridge	(3, 2)	2.439 (±0.570)
	Centre	Ridge	(3, 0.001)	2.188 (±0.755)
	Full	Ridge	(4, 0.1)	1.479 (±0.580)
	Subject huge	Ridge	(3, 0.001)	2.523 (±0.771)
	Subject small	SVR	(‘linear’, 1, 0.01, 0.0001)	4.678(±0.430)
0.005	5-point	Lasso	(3, 0.001)	2.908 (±1.104)
	9-point	Ridge	(3, 1)	2.215 (±0.841)
	Centre	Ridge	(4, 0.01)	2.297 (±0.674)
	Full	Ridge	(5, 1)	1.490 (±0.508)
	Subject huge	Ridge	(3, 0.01)	2.555 (±0.687)
	Subject small	SVR	(‘linear’, 1, 0.01, 0.0001)	4.806 (±0.441)
0.010	5-point	Lasso	(3, 0.001)	2.979 (±1.286)
	9-point	Ridge	(3, 1)	2.379 (±0.667)
	Centre	Ridge	(3, 0.001)	2.478 (±0.901)
	Full	Ridge	(5, 0.1)	1.731 (±0.622)
	Subject huge	Ridge	(3, 0.01)	2.791 (±0.822)
	Subject small	SVR	(‘poly’, 1, 0.01, 0.001)	5.048 (±0.378)
0.050	5-point	SVR	(‘linear’, 1, 0.1, 0.1)	4.346 (±0.502)
	9-point	Ridge	(4, 2)	3.943 (±0.566)
	Centre	SVR	(‘poly’, 1, 0.1, 0.001)	4.370 (±0.312)
	Full	SVR	(‘rbf’, 1, 0.1, 0.001)	3.285 (±0.357)
	Subject huge	SVR	(‘linear’, 1, 0.01, 0.001)	4.361 (±0.311)
	Subject small	RANSAC	(1,)	8.313 (±0.727)

## Evaluations

In this section we discuss our results with the simulated gaze vectors and start with an overview of all methods used, followed by a closer look at the polynomial and ridge regression (as polynomial approaches are commonly used for calibration). More specifically, we look at the error within and outside the calibration range is considered and at the end the results of the experiment and the corresponding simulation are shown. For all statements, we report the p-value of the t-test or the F-value with corresponding p-value to determine if the difference is statistically significant.

### Comparative view on all methods

**Figure 6.**
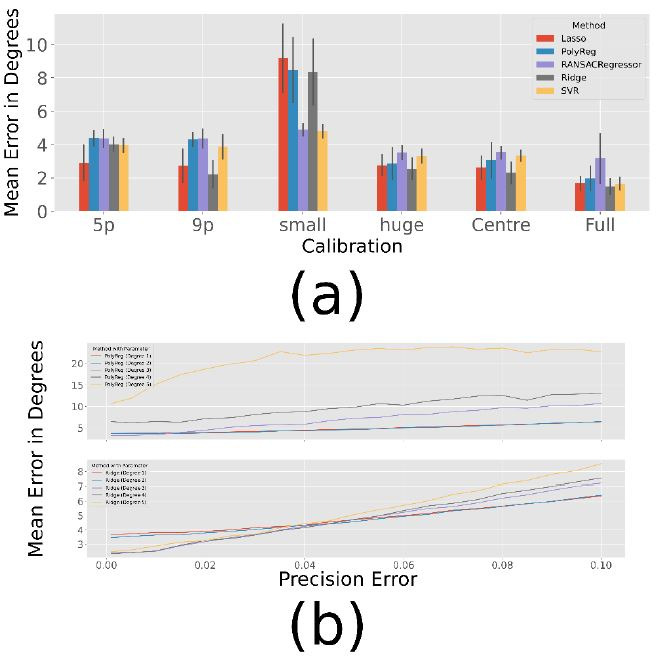
Results over 100 Simulations with the parameters: $p_{error}=0.1,\;r_{error}=0.5$. (a) Best Results with fixed $\sigma_{prec}=0.005$. (b) Best Results for polynomial (top) and ridge (bottom) regression for different precision errors and the centre calibration (Figure 4 (c)).

Before assessing the performance of individual methods in more detail, we compared all methods against each other. For each method, several parameters (such as the degree for the estimated polynomial) are tested in a grid-search based approach. The best results for each calibration pattern are shown in Figure 6 (a), where the parameters of the simulation are set to: $p_{error}=0.1,\;r_{error}=0.5,\;\sigma_{prec}=0.005$ (we found these to match our real-world data). It can be seen that the choice of calibration pattern has a significant impact on the performance of the method. To prove this, we perform ANOVA for each method: polynomial regression ( F=434,p<0.001), ridge regression ( F=610,p<0.001), lasso regression ( F=569,p<0.001), RANSACRegressor ( F=76,p<0.001) and SVR ( F=445,p<0.001). In the following, these differences are examined in more detail.

The calibration with 5 and 9 points works equally well for the polynomial regression, RANSACRegressor, Lasso and SVR ( p>0.05). But for the Ridge method, the extra 4 points seem to improve performance significantly ( p<0.001). The centre calibration seems to lead to slightly better results than the 9-point calibration ( p<0.001 for all methods expect Lasso and Ridge with p>0.05). It can be assumed that the estimation within the calibrated range works better with the central calibration, but outside of this range the 9-point calibration is less likely to lead to overfitted polynomials that explode when leaving the calibrated area. This explains why the full calibration works better then centre ( p<0.001 for all methods except RANSAC with p=0.011), because in the small range where the centre calibration does not fit, the full calibration gives much better results and compared to the 9-point calibration ( p<0.001 for all methods) there is much more data for a better estimate.

The problem with extrapolation is also evident in the small calibration pattern, for which all methods lead to a rather poor result. Even the 5-point calibration gives better results ( p<0.001) for each method. The huge subject pattern, on the other hand, seems to lead to similar results as the centre pattern for the most methods ( p>0.05 for all methods except of Ridge with p=0.008). Only Ridge performs slightly better on with the centre pattern ( p=0.004).Since it covers almost the same part of the field, this was to be expected.

The best results for different precision qualities are shown in Table 3. As expected, the error increases as the precision decreases. In most cases, however, the ridge regression gives the best results, except for the small subject pattern, which seems to cover too small a range to estimate a good polynomial. This could explain why the only method that does not estimate a polynomial, usually leads to the best results. Furthermore, Figure 6 (b) shows the course of the polynomial and ridge regression for increasing precision errors in the centre calibration. In addition, graphics for the other methods can be found in the appendix. As shown in the figure, the higher degrees of ridge regression work better for very small errors, while they are still slightly better for high errors.

In summary, most methods work well, with a slight advantage for ridge regression. Only when the calibration fits a very small range do the SVR and the RANSACRegressor outperform the other methods. However, the RANSACRegressor seems to perform worst in all other cases.

### Polynomial Regression

**Figure 7.**
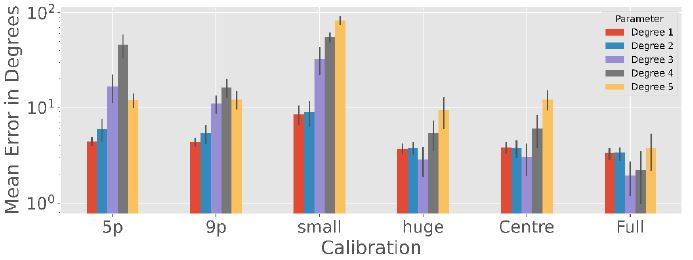
Polynomial Regression accuracy with an exponential scale averaged over 100 simulation runs for the different calibration patterns. Clearly, higher polynomial degrees require a more complex and complete calibration procedure, while degrees above three are unlikely to yield good results due to overfitting the calibration data.

Since polynomial regression is often used in calibration scenarios, we will examine this method in more detail as shown in Figure 7.

There are significant differences in the performance of the degree used for the estimated polynomial in the 5-point ( F=658,p<0.001), 9-point ( F=429,p<0.001), subject small ( F=1847,p<0.001), subject huge ( F=200,p<0.001), centre ( F=442,p<0.001) and full ( F=57,p<0.001) calibration patterns.

When the calibration range is large, such as the centre, full and subject huge pattern, the polynomial with degree 3 seems to be the best choice ( p<0.001 except full calibration with degree 3 and 4 with p=0.046). However, if there is only one viewpoint or the calibration is only in the middle (Subject small), degree 3 or higher seem to overfit, so grade 1 and 2 are better ( p<0.001). However, there is no significant difference in the subject small calibration between degree 1 and 2 ( p>0.05).

### Ridge Regression

**Figure 8.**
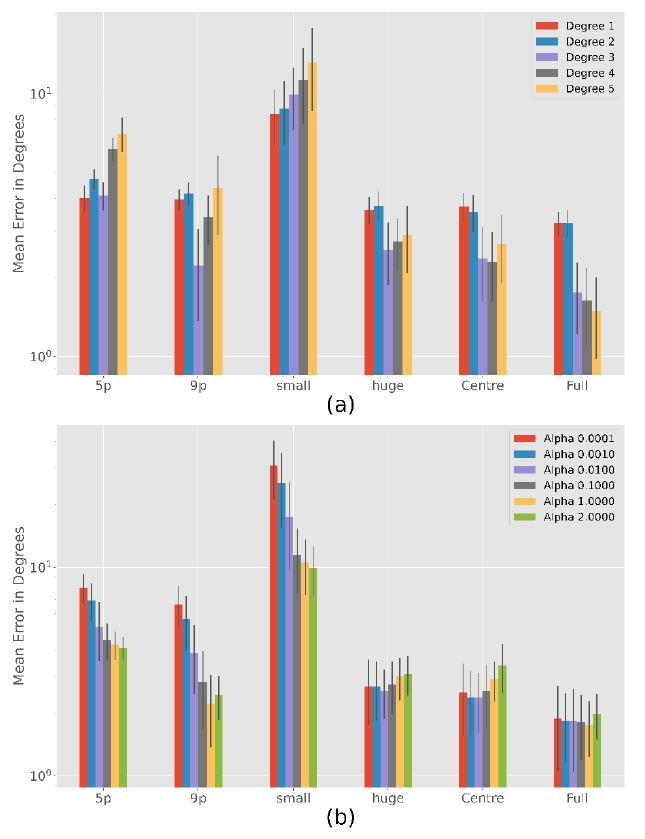
Ridge Regression accuracy with an exponential scale over 100 simulation runs. Results are relatively stable for different degrees with a possible optimum at degree three. The more complete the calibration, the less important is the choice of a good α. (a) Analysis of the polynomial degree over different calibration patterns, with the respective alpha that led to the best result. (b) Influence of the α parameter.

Since the ridge regression leads to the best results for most calibration patterns (see Table 3) we will take a closer look at it. Figure 8 (a) shows the best results of the different degrees of the polynomials. Like polynomial regression there are significant differences between the chosen degree in the 5-point ( F=403,p<0.001), 9-point ( F=101,p<0.001), subject small ( F=37,p<0.178), subject huge ( F=73,p<0.001), centre ( F=103,p<0.001) and full ( F=352,p<0.001) calibration patterns. In the following, we will take a closer look at where the significant differences are.

Degree 1 and 2 results are very similar for each calibration ( p>0.05 except of 5-point calibration with p<0.001). Degree 3, 4, and 5 are usually better as the number of calibration points increases ( p<0.001), except for Degree 3 where no significant differences in performance between 9 point and centre calibration can be found ( p>0.05) and 9 point is slightly better than subject huge ( p<0.001). Calibration Subject small results in the largest error for each degree ( p<0.001).

Since degree 3 is usually one of the best, Figure 8 (b) shows the different results of degree 3 with different weighting parameters. There are significant differences between the chosen alphas in the 5-point ( F=189,p<0.001), 9-point ( F=209,p<0.001), subject small ( F=158,p<0.001), subject huge ( F=6.7,p<0.001) and centre ( F=23,p<0.001). No significant difference is found between alphas for the full calibration ( F=1.3,p>0.05). As the figure shows, a larger alpha lead to better results ( p<0.001) for a small number of calibration points (5- and 9-point calibration) or a small calibration area (subject small). Only in the 5-point calibration and subject small for alpha equal to 1 and 2 there is no statistically significant difference ( p>0.05). In the case of Subject huge and Centre calibration, there are no significant differences between α=0.0001,…,0.1 ( p>0.05). Only the error for alpha equal to 1 or 2 is significantly larger than for the lower alphas ( p<0.001).

In summary, we can say that α=0.01 is a good choice in the most cases.

### Intra- and Extrapolation

**Figure 9.**
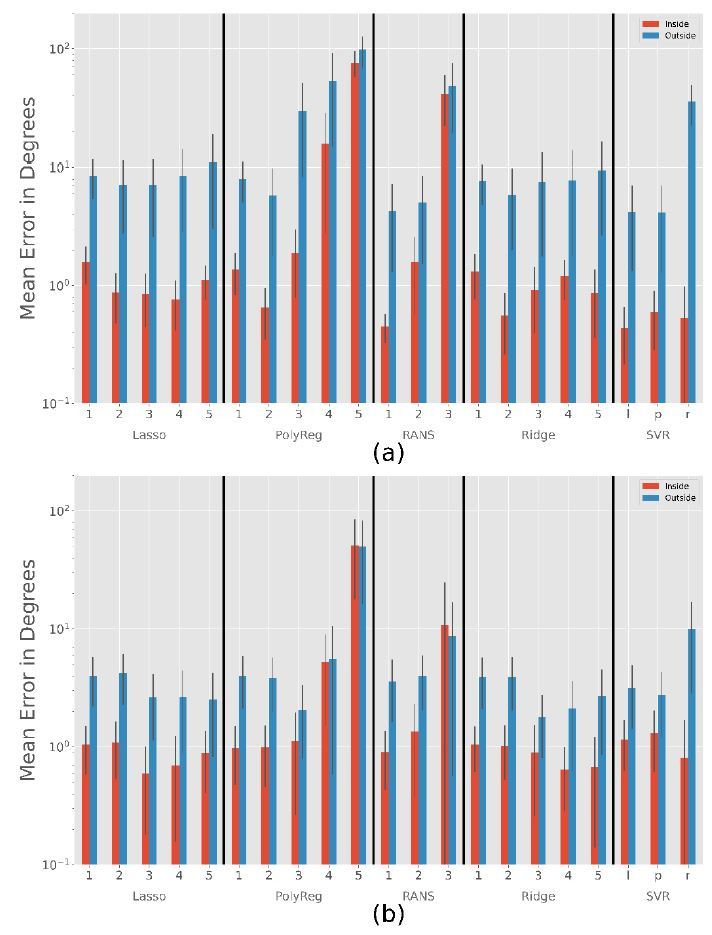
Results for all methods with closer look at the different error inside and outside the calibration range depending on the use of the subject small or large calibration pattern. RANS stands for RANSAC, l for linear, p for poly and r for rbf. (a) Subject small (b) Subject huge. Note the exponential scale.

Figure 9 shows the average error occurring during interpolation and extrapolation of the calibration range. While the large coverage calibration pattern (Figure 4 €) has only a few points outside this range, the small pattern (Figure 4 (f)) has many points outside. Shrinkage methods (ridge and lasso regression) work relatively well in both cases. Note that these methods have an α parameter to prevent overfitting. As a consequence, increasing the polynomial’s degree does not have a negative impact. Figure 9 shows the best results achieved for both cases. For the large range, a small alpha value such as α=0.1 and for the small range a large alpha value such as α=2 could be used instead of changing the degree. However, it seems better to decrease the degree instead of increasing the alpha, as shown in Table 4.

The polynomial regression works as expected: If the degree is too high, the method adjusts too accurately to the calibration points and does not eneralize well for other data. The same problem occurs with the RANSACRegressor. The SVR has the most parameters, but even if we use the one that leads to the smallest extrapolation error, it is only slightly better than the ridge regressor when calibrating only on a small area. Moreover, it performs worse on the large calibration pattern.

**Table 4.** Best extrapolation results of each method for the calibration patterns Subject small and huge with their corresponding parameters. The standard deviation is given in the parentheses.

**Method**	**Calibration**	**Parameter**	**Inside (°)**	**Outside (°)**
Lasso	Subject small	(2, 0.0001)	0.879 (±0.401)	7.112 (±4.348)
PolyReg	Subject small	(2,)	0.657(±0.306)	5.849 (±4.054)
RANSAC	Subject small	(1,)	**0.452** (±0.126)	4.270 (±2.960)
Ridge	Subject small	(2, 0.0001)	0.562 (±0.302)	5.930 (±3.918)
SVR	Subject small	(‘poly’, 1, 0.01, 0.001)	0.596 (±0.310)	**4.177** (±2.886)
Lasso	Subject huge	(5, 0.001)	**0.888** (±0.480)	2.537 (±1.716)
PolyReg	Subject huge	(3,)	1.112 (±0.847)	2.070 (±1.275)
RANSAC	Subject huge	(2,)	0.904 (±0.468)	3.588 (±1.964)
Ridge	Subject huge	(3, 0.0001)	0.900 (±0.641)	**1.789** (±0.985)
SVR	Subject huge	(‘poly’, 1, 1, 0.0001)	1.320 (±0.707)	2.770 (±1.572)

### Experimental Results

**Figure 10.**
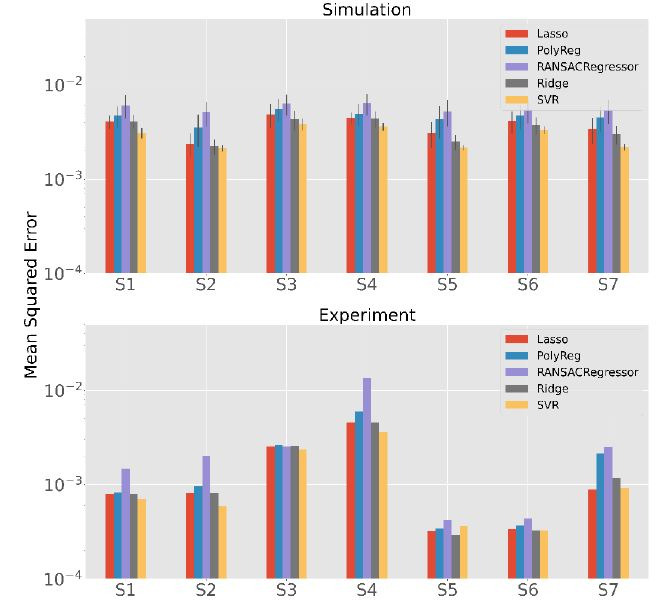
Comparison of the results of the simulation and the real-world experiment with an exponential scale of the MSE.

Figure 10 shows the results of the experiment (bottom) and the simulation with the same calibration and test patterns as the experiment (top). Note that in the case of the simulation, 100 iterations were performed for each method and subject sample, while in the real experiment the subject only performs one calibration and one validation. For this reason, we can see the error bars created only for the simulation. In both scenarios, the methods have similar results for the different subjects. The most striking observation on the real data is that the mean squared error (MSE) for Subjects S5 and S6 is clearly lower than for the other subjects. There are two possible explanations for this. The first is that the measurement errors in the extracted gaze vectors are lower than for the others. The second is that the calibration and test patterns are more like each other. Since this effect does not occur in the simulation scenario, the first reason is more likely because a minor error rate in individual participants is possible, while it is very unlikely that only small errors occur in 100 simulations.

Furthermore, the performance of the SVR seems to be better than that of the other methods, this can be seen especially in the simulation ( p<0.001 except of Ridge for Subject 2 with p=0.004). This is a different result than in the previous analyses. One possible reason for this is that the calibration patterns are very similar to the test patterns due to the design of the experiment.

With the exception of SVR, ridge regression seems to outperform the other methods mostly ( p<0.001). In terms of numbers, the mean MSE of the ridge regression is for the simulation about 20% better than that of the usually used polynomial regression which is a significance improvement ( p<0.001). In the real-world experiment, it is on average about 15% better. The differences are quite small in S1 and S3, but they are clearly visible in the others.

## Limitations

In this work, we compared the simulated Gaze Vectors with Look's system. Accordingly, the systematic error was also created. If a different system is used, the systematic error may also change. This must then be added to the error free Gaze Vectors like the systematic error used in this work. In addition, we compared the simulated gaze vectors with real gaze vectors recorded in calibration sessions in a room. This way, erroneous measurements that can occur, for example, due to lighting conditions or because the participants do not follow the target properly, are not taken into account. Only the systematical, mismatching and the noise error are relevant in our simulation, which we found in our real data.

Another limitation is that the real-world study has only seven participants. This is too few to be able to make statistical statements, which is why in this work they only serve as a proof of concept for the transferability of the simulation results to the real world.

## Conclusions

In this paper, the focus is on looking at different methods for mapping measured gaze vectors onto the scene video of a mobile eye tracker. For a simulation of gaze vectors, we proposed different types of noise and measurement errors, such as decreased overall precision, location dependent precision loss as well as false pupil detection. This allowed us to examine the four methods of polynomial, lasso, ridge and support vector regression to see how well they perform under different mixtures of noise.

Overall, ridge regression robustly showed good results over a broad spectrum of parameters, especially if the precision error of the eye tracker is not too large and the calibrated area not too small. In addition, we observed that the ridge regression does not suffer in accuracy when the degree of the polynomial is increased, as can be observed for normal polynomial regression.

By adjusting the simulation parameters to match the expected real-world data characteristics, the optimal mapping function for a specific device or specific recording conditions can be determined.

In this work, we focus on head-mounted eye trackers, so the evaluation mainly discusses the results of Smooth Pursuit calibration patterns. However, this simulation approach could also work for remote eye trackers, which often use n-point calibrations. For example, Figure 6 shows that the results for Ridge and Lasso are also good.

### Acknowledgments

We acknowledge support from the Open Access Publication Fund of the University of Tübingen.

## Figures and Tables

**Figure 11.**
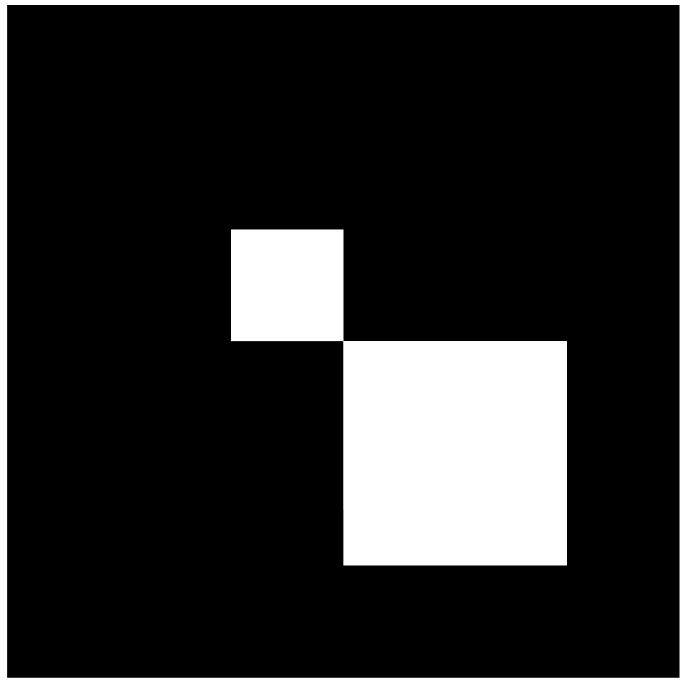
Example of an aruco-marker.

**Figure 12.**
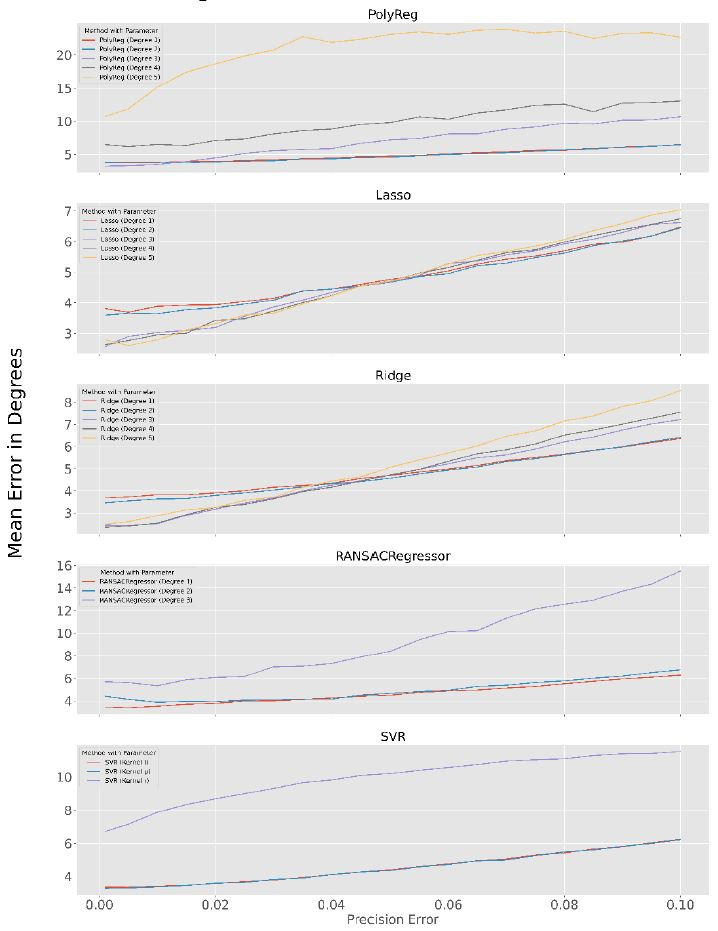
The diagrams show the course of the error with increasing precision error. Except for the parameters indicated in the legend, the parameters with the best results are used.

## Appendix

### Calculation of the error in degrees

The target and estimate points $p_{true},\;p_{est}\in \;[0,\;1{]}^{2}$ give the proportion of the horizontal and vertical axis to determine where the point is in the camera's image. The following describes how to calculate the angle between the true point and the estimated point. The coordinates of the camera $c\in \;{\mathbb{R}}^{3}$, the distance d∈ℝ to the plane in which the viewpoint moves, the x-coordinates on the plane of the left and right image edge $x_{l},\;x_{r}\in \;\mathbb{R}$ and the y-coordinates on the plane of the top and bottom image edge $y_{t},\;y_{b}\;\in \;\mathbb{R}$ are also known. The formula t is now given as:

$$t\left(p\right)=\;\left(\begin{array}{c} p_{x}\cdot d_{h}+x_{l}\\ p_{y}\cdot d_{v}+y_{b}\\ d+c_{z} \end{array}\right)$$

Here $d_{h}\;=\;x_{r}\;-\;x_{l}$ is the distance between the left and right edge of the image on the plane, $d_{v}\;=\;y_{t}\;-\;y_{b}$ is the distance between the bottom and the top, $c_{z}$ is the z-coordinate of the camera and $p_{x}$ and $p_{y}$ are the x- and y-coordinates of the point to be transformed.

With this, the direction vectors $d_{true},\;d_{est}\in {\mathbb{R}}^{3}$ from the camera position to the points $p_{true}$ and $p_{est}$ can be calculated:

$$d_{true}=t\left(p_{true}\right)-c$$

$$d_{est}=t\left(p_{est}\right)-c$$

And now the angle $\alpha_{error}$ between the true and the estimated point is given:

$$\alpha_{error}=arccos\left(\frac{d_{true}^{T}\cdot d_{est}}{{∥d_{true}∥}_{2}\cdot {∥d_{est}∥}_{2}}\right)$$

### Unity

Unity is a cross-platform game engine and development environment. It provides a large toolkit for 2D and 3D simulation and game programming. In each scene of the project, there is usually a camera object that defines the player's point of view. In the case of this work, this is the scene camera, which we need to define where the viewpoint must be positioned to match the desired pattern from the scene camera's point of view. In contrast, the view of the eye cameras are not needed, so we use cubes as dummies to calculate the necessary angles for the gaze vectors using functions from Unity. In content of this work the important things are:

- Create a scene by drag-and-drop of game objects like spheres and cubes.
- Manipulate the scene using C#-scripts.
- Easily switch between the world coordinate system and the camera’s point of view.
- Efficient calculation and extraction of data with unity's integrated functions.

### Aruco-marker

An Aruco marker is an easy to detect image with a black border. The inner consists of black and white squares. The black border facilitates its fast detection in the image. Figure 11 shows an example of an aruco-marker.

### Influence of the precision error to the Methods

Here are diagrams for all methods used, showing the influence of the precision error on the mean error.

## References

[b1] Atchison, D. A. (2017). Axes and angles of the eye, volume one. In P. Artal (Ed.), Handbook of visual optics (pp. 455–467). CRC Press., 10.1201/9781315373034

[b2] Blignaut, P. (2014, January). Mapping the Pupil-Glint Vector to Gaze Coordinates in a Simple Video-Based Eye Tracker. Journal of Eye Movement Research, 7. 10.16910/jemr.7.1.4 1995-8692

[b3] Blignaut, P. (2016). Idiosyncratic feature-based gaze mapping. Journal of Eye Movement Research, 9. 10.16910/jemr.9.3.2 1995-8692

[b4] Cortes, C., & Vapnik, V. (1995). Support-vector networks. Machine Learning, 20, 273–297. 10.1007/BF00994018 0885-6125

[b5] Drewes, H., Pfeuffer, K., & Alt, F. (2019). Time- and Space-Efficient Eye Tracker Calibration. Proceedings of the 11th ACM Symposium on Eye Tracking Research & Applications. New York, NY, USA: Association for Computing Machinery. 10.1145/3314111.3319818

[b6] Drucker, H., Burges, C. J., Kaufman, L., Smola, A., & Vapnik, V. (1997). Support vector regression machines. Advances in Neural Information Processing Systems, 9, 155–161.1049-5258

[b7] Fischler, M. A., & Bolles, R. C. (1981, June). Random Sample Consensus: A Paradigm for Model Fitting with Applications to Image Analysis and Automated Cartography. Communications of the ACM, 24, 381–395. 10.1145/358669.358692 0001-0782

[b8] Fuhl, W., Schneider, J., & Kasneci, E. (2021). 1000 Pupil Segmentations in a Second using Haar Like Features and Statistical Learning. International Conference on Computer Vision Workshops, ICCVW. 10.1109/ICCVW54120.2021.00386

[b9] Hassoumi, A., Peysakhovich, V., & Hurter, C. (2019). Improving eye-tracking calibration accuracy using symbolic regression. PLoS One, 14, e0213675. 10.1371/journal.pone.0213675 1932-6203 30875387 PMC6420251

[b10] Hoerl, A. E., & Kennard, R. W. (1970). Ridge regression: Biased estimation for nonorthogonal problems. Technometrics, 12, 55–67. 10.1080/00401706.1970.10488634 0040-1706

[b11] Kasprowski, P., Harezlak, K., & Stasch, M. (June 2014). Guidelines for eye tracker calibration using points of regard., 284. 10.1007/978-3-319-06596-0_21

[b12] Kim, J., Stengel, M., Majercik, A., De Mello, S., Dunn, D., Laine, S., . . . Luebke, D. (2019). Nvgaze: An anatomically-informed dataset for low-latency, near-eye gaze estimation. Proceedings of the 2019 CHI Conference on Human Factors in Computing Systems, (S. 1–12). 10.1145/3290605.3300780

[b13] Kübler, T. C. (December 2021). Look! Blickschulungsbrille: Technical specifications. Tech. rep., Look! ET.

[b14] Nair, N., Kothari, R., Chaudhary, A. K., Yang, Z., Diaz, G. J., Pelz, J. B., & Bailey, R. J. (2020). RIT-Eyes: Rendering of near-eye images for eye-tracking applications. ACM Symposium on Applied Perception 2020, (S. 1-9). 10.1145/3385955.3407935

[b15] Narcizo, F. B., Dos Santos, F. E. D., & Hansen, D. W. (2021). High-Accuracy Gaze Estimation for Interpolation-Based Eye-Tracking Methods. Vision, 5, 41. 10.3390/vision5030041 2411-5150 34564339 PMC8482219

[b16] Pedregosa, F., Varoquaux, G., Gramfort, A., Michel, V., Thirion, B., Grisel, O., . . . Duchesnay, E. (2011). Scikit-learn: Machine Learning in Python. Journal of Machine Learning Research, 12, 2825–2830.1532-4435

[b17] Santini, T., Fuhl, W., Geisler, D., & Kasneci, E. (February 2017). EyeRecToo: Open-Source Software for Real-Time Pervasive Head-Mounted Eye-Tracking. 12th Joint Conference on Computer Vision, Imaging and Computer Graphics Theory and Applications (VISIGRAPP 2017). 10.5220/0006224700960101

[b18] Santini, T., Niehorster, D. C., & Kasneci, E. (June 2019). Get a Grip: Slippage-Robust and Glint-Free Gaze Estimation for Real-Time Pervasive Head-Mounted Eye Tracking. Proceedings of the 2019 ACM Symposium on Eye Tracking Research & Applications (ETRA). 10.1145/3314111.3319835

[b19] Tibshirani, R. (1996). Regression Shrinkage and Selection via the Lasso. Journal of the Royal Statistical Society. Series B. Methodological, 58, 267–288. 10.1111/j.2517-6161.1996.tb02080.x 0035-9246

[b20] Wood, E., Baltrušaitis, T., Morency, L.-P., Robinson, P., & Bulling, A. (2016). Learning an appearance-based gaze estimator from one million synthesised images. Proceedings of the Ninth Biennial ACM Symposium on Eye Tracking Research & Applications, (S. 131–138). 10.1145/2857491.2857492

